# Core RNA Interference Genes Involved in miRNA and Ta-siRNA Biogenesis in Hops and Their Expression Analysis after Challenging with *Verticillium nonalfalfae*

**DOI:** 10.3390/ijms22084224

**Published:** 2021-04-19

**Authors:** Urban Kunej, Jernej Jakše, Sebastjan Radišek, Nataša Štajner

**Affiliations:** 1 Department of Agronomy, Biotechnical Faculty, University of Ljubljana, 1000 Ljubljana, Slovenia; urban.kunej@bf.uni-lj.si (U.K.); jernej.jakse@bf.uni-lj.si (J.J.); 2 Plant Protection Department, Slovenian Institute of Hop Research and Brewing, 3310 Žalec, Slovenia; sebastjan.radisek@ihps.si

**Keywords:** *Humulus lupulus*, *Verticillium nonalfalfae*, fungal infection, RNA interference, plant–pathogen interactions, small RNA

## Abstract

RNA interference is an evolutionary conserved mechanism by which organisms regulate the expression of genes in a sequence-specific manner to modulate defense responses against various abiotic or biotic stresses. Hops are grown for their use in brewing and, in recent years, for the pharmaceutical industry. Hop production is threatened by many phytopathogens, of which *Verticillium*, the causal agent of Verticillium wilt, is a major contributor to yield losses. In the present study, we performed identification, characterization, phylogenetic, and expression analyses of three Argonaute, two Dicer-like, and two RNA-dependent RNA polymerase genes in the susceptible hop cultivar Celeia and the resistant cultivar Wye Target after infection with *Verticillium nonalfalfae*. Phylogeny results showed clustering of hop RNAi proteins with their orthologues from the closely related species *Cannabis sativa*, *Morus notabilis* and *Ziziphus jujuba* which form a common cluster with species of the Rosaceae family. Expression analysis revealed downregulation of argonaute 2 in both cultivars on the third day post-inoculation, which may result in reduced AGO2-siRNA-mediated posttranscriptional gene silencing. Both cultivars may also repress ta-siRNA biogenesis at different dpi, as we observed downregulation of argonaute 7 in the susceptible cultivar on day 1 and downregulation of RDR6 in the resistant cultivar on day 3 after inoculation.

## 1. Introduction

Post-transcriptional gene silencing (PTSG), or RNA interference (RNAi), is considered an evolutionarily conserved mechanism since it has been found across all main eukaryotic lineages and is involved in many biological processes [1]. Small non-coding RNAs (sRNAs), namely small interfering RNAs (siRNAs) or microRNAs (miRNAs), have the central role in dictating the RNAi process and are classified based on their biogenesis, precursor structure and mode of action. While miRNAs are derived from single-stranded precursors with a hairpin or stem-loop structure, siRNAs are produced from longer double-stranded RNAs (dsRNAs) [2]. Despite their differences, the mode of action of both classes of sRNAs converges in utilization of the RNAi machinery, i.e., proteins that guide gene silencing, and play an important role in the defense mechanism of plants against various biotic and abiotic stresses [3]. The RNA silencing mechanism is initiated by Dicer-like proteins (DCL) that cleave long dsRNAs into short double-stranded siRNA and hairpin-structured miRNA precursors into miRNA duplexes (composed of miRNA and miRNA star-miRNA*-derived from the opposite arm in the precursor). The generated fragments are 20–24 base pairs long and have two nucleotide overhangs at the 3′ end. All DCLs consist of a DExD/H-box helicase domain, a centrally positioned PAZ domain (named after the Piwi, Argonaut and Zwille proteins), followed by two ribonuclease III (RNase III) catalytic domains and a double-stranded RNA-binding domain/motif [4]. In plants, DCL1 produces 21–24 bp long miRNA duplexes from imperfectly paired hairpin RNAs, transcripts of miRNA genes [5], DCL2 produces siRNA from virus-derived RNAs, DCL3 cleaves dsRNAs generated by RDR2 into siRNAs and is involved in transcriptional silencing of repeat DNA and transposons by DNA methylation, and DCL4 is involved in the production of 21 bp long trans-acting small interfering RNAs (ta-siRNAs) [6]. Ta-siRNA biogenesis is directed by specific miRNAs that associate with argonaute 7 (AGO7) and cleave transcripts of *TAS* genes. The resulting fragments are reverse transcribed by RNA-dependent RNA polymerase 6 (RDR6) and processed by Dicer-like 4 (DCL4) in 21 nt phasing to produce 21 nt long ta-siRNAs [7,8]. After processing by DCLs, sRNAs are loaded into Argonaute proteins (AGOs), which are major catalytic components of the RNA-induced silencing complex (RISC). The sRNA embedded in AGO serves as a template for RISC to recognize a nearly complementary sequence in the messenger RNA (mRNA) and perform sequence-specific regulation of gene expression. Plants encode different AGO proteins that have different functions and bind specific sRNAs and are therefore involved in different RNAi pathways. All AGO proteins are comprised of three main functional and evolutionarily conserved domains; the PAZ, Middle (MID) and P-element induced wimpy (PIWI) domains. The latter domain functions as an RNase H endonuclease and is responsible for RNA cleavage [9]. Phylogenetic analyses of plant AGO proteins classified them into three major clades; AGO1/5/10, AGO2/3/7, and AGO4/6/8/9 [10]. However, their evolutionary relationship does not reflect their functional similarity. For example, AGO4, AGO6, and AGO9 bind 24-nt-long sRNAs, whereas AGO1, AGO2, and AGO7 bind 21- to 22-nucleotide sRNAs (either miRNAs or ta-siRNAs) [10]. Additionally, in *Arabidopsis*, AGO1 preferentially binds miRNAs with a length of 21 nucleotides and recruits small RNAs with a 5’ terminal uridine [11] but also binds miRNAs with a length of 22 nucleotides to trigger RDR6-dependent biogenesis of secondary siRNAs, e.g., ta-siRNAs [8]. AGO7 is involved in the RDR6/SGS3/DCL4/AGO7 trans-acting siRNA pathway. It associates mainly with microRNA390 (miR390), which guides the cleavage of *TAS3* transcripts. Both AGO2 and AGO7 preferentially bind small RNAs that have a 5’ terminal adenosine and are 21 nucleotides long [12,13].

Hops (*Humulus lupulus* L.) are traditionally cultivated for the use in brewing industry, for the characteristic flavor and aroma, and as a stabilizer and preservative. In recent years, hops have also been used for pharmaceutical purposes, as many beneficial health effects have been noted [14,15]. Plant pests and diseases are responsible for 20–40% of the worldwide crop losses of economically important crops [16]. Fungal diseases are particularly dangerous, especially those caused by soil-borne pathogens such as *Verticillium nonalfalfae* and *Verticillium dahliae*. Verticillium wilt disease occurs worldwide and attacks both annual crops and perennial woody plants, affecting food and feed production and threatening natural ecosystems. Symptoms of Verticillium wilt of hops caused by *V. nonalfalfae* vary with the pathogenicity of the strain. Mild wilting symptoms may be observed when infected with a mild pathotype of the fungus, but infection with the lethal pathotype may result in plant death or destruction of the entire plantation [17,18]. In response to invading pathogens, plants have evolved multi-tiered immune response in which RNAi plays an important role by controlling the sequence-specific regulation of the gene expression and processing of genetic material of pathogens [19]. RNAi components have also been described in fungal species and have a similar function to those found in plants [20,21,22]. Recent studies on RNAi have shown that small RNAs involved in gene silencing processes can be transferred bidirectionally between plant pathogens and their hosts. This phenomenon is known as cross-kingdom RNAi and represents a form of communication between two interacting organisms, such as the plant and its pathogen. RNAi signals, in particular miRNAs protected in extracellular vesicles, can be transmitted through the plant by phloem flow [23]. Zhang et al. [24] discovered two cotton miRNAs, miR166 and miR159, that are in response to infection with *V. dahliae* exported to fungal hyphae, where they trigger silencing of genes responsible for fungal pathogenicity. On the other hand, small RNAs produced by fungal DCL can enter host cells and bind to host AGO proteins to inhibit pathogenesis-related genes involved in plant defense mechanisms [25]. However, host-pathogen interactions mediated by RNAi components appear to be specific. While *Arabidopsis ago1* and *ago2* mutants showed increased susceptibility to the necrotrophic fungus *Sclerotinia sclerotiorum* and overexpression of *AGO1* increased resistance [26], *Arabidopsis ago1* mutants showed reduced susceptibility to *Botrytis cinerea* [25]. In addition, *Arabidopsis dcl2*, *dcl4*, *rdr6*, *rdr2*, and *ago7* mutants were found to be more susceptible and *ago1* mutants more resistant to *V. dahliae* compared to inoculated Col-0 plants [27].

In our research, we investigated interactions between susceptible or resistant hop plants and *Verticillium nonalfalfae* at the level of hop RNAi components. We focused on RNAi components involved in miRNA biogenesis, i.e., DCL1, and miRNA-binding AGO proteins which are involved in PTGS (AGO1, AGO2). We also examined core components of secondary siRNA biogenesis; RDR2, RDR6 and AGO7. To elucidate the functions of RNAi components in the early phase of Verticillium wilt pathogenesis, their identification and characterization, expression patterns, and phylogenetic relationships were investigated.

## 2. Results

### 2.1. Characterization and Structural Analysis of RNAi Genes

Using BLASTX analysis and retrieved sequences of DCL, AGO and RDR proteins from the UniProtKB database, we identified their homologs in the hops’ draft genome and transcriptome. Additionally, gene models of DCL, AGO and RDR in the hop genome were manually curated based on RNA sequencing data (RNA-Seq) [28] and BLAST results. Thus, we identified and built gene models for *DCL1* (MW658774), *DCL4* (MW658775), *AGO1* (MW658771), *AGO2* (MW658772), *AGO7* (MW658773), *RDR2* (MW658776), and *RDR6* (MW658777) in hops genome. The features of the individual genes are shown in Table 1.

**Figure 1:**
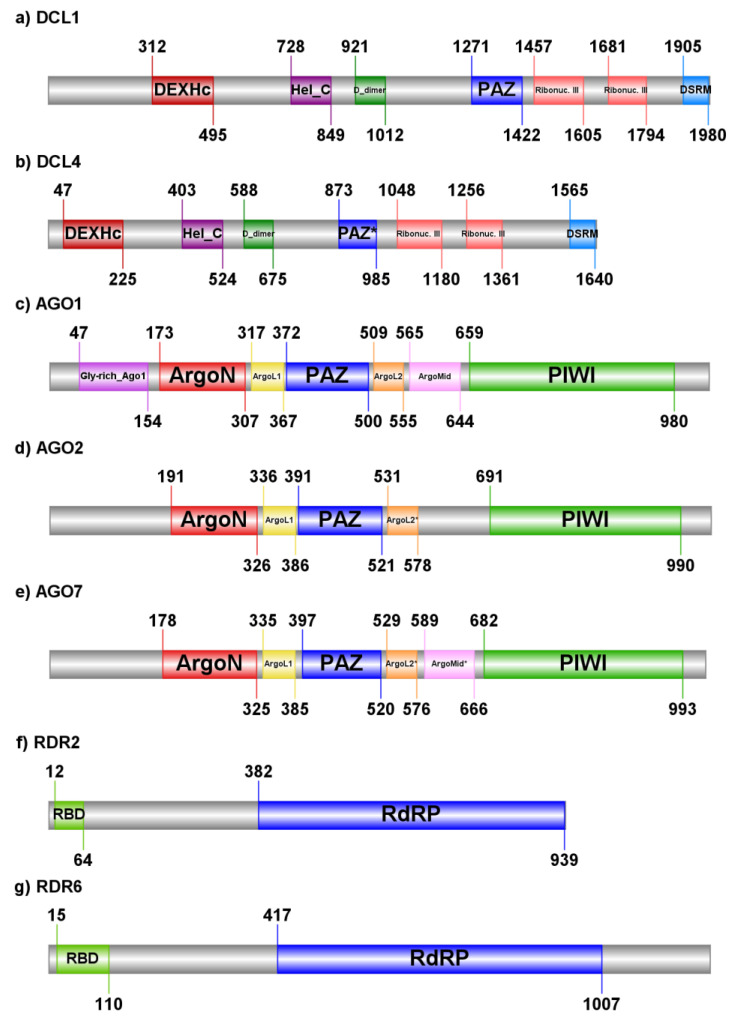
Protein domains identified in seven hop’s RNAi proteins. (**a**,**b**) Hop DCL proteins contain DEXHc-DEXH-box helicase domain of endoribonuclease Dicer at N-terminal part, Hel_C-Helicase-C domain, D_dimer-Dicer dimerisation domain, Ribonuc. III-ribonuclease III domain, and DSRM-double stranded RNA binding motif. (**c**–**e**) Domain analysis of AGO proteins shows the presence of Gly-rich_Ago1-Glycine-rich region of Argonaut, ArgoN-N-terminal domain of argonaute, ArgoL1-Argonaute linker 1 domain, PAZ–Piwi-Argonaute-Zwille domain, ArgoL2-Argonaute linker 2 domain, ArgoMid-Mid domain of argonaute and PIWI–P-element induced wimpy domain. (**f**,**g**) RDR proteins encompass RBD–RNA binding domain and RdRP–RNA-dependent RNA polymerase. “*” in the domain name denotes domains with less significant matches to the Pfam database, but which have also been identified with Conserved Domain Database (CDD). Domains were visualized with the online tool “llustrator of Biological Sequences” (IBS; http://ibs.biocuckoo.org/online.php; accessed on 16 April 2021).

The *DCL1* gene of hops contains 19 introns and encodes transcripts with a length of 6497 nt. The coding sequence of the transcripts has a length of 1984 amino acids. The same number of introns can be observed in *DCL1* of hop closely related species *Cannabis sativa* (XM_030633569.1) and in the model organism *A. thaliana* (NM_099986.4), with which the hop DCL1 protein sequence shares 92.67% identity at 99% coverage and 73.97% identity at 100% coverage, respectively. Compared to the hop DCL1, the DCL4 transcript is shorter (5281 nt) and encodes a protein with a length of 1645 amino acids. However, the *DCL4* gene contains 24 introns. At the protein level, hops DCL1 and DCL4 have a 31.1% identity, while no significant similarity can be found at the nucleotide level. Both hops DCL proteins, DCL1 and DCL4, contain a type III restriction enzyme domain (ResIII; PF04851) or a so-called DEXH-box helicase domain of endoribonuclease Dicer at the N-terminal part (DEXHc_dicer; cd18034), followed by a helicase-C domain (Helicase_C; PF00271), a Dicer dimerization domain (PF03368), a PAZ domain and two ribonuclease III domains (PF00636) as well as a double strand RNA binding domain from DEAD END PROTEIN 1 (DND1_DSRM; PF14709) at C-terminal part of the amino acid sequence (Figure 1a,b). Using a conserved domain database (CDD), we identified ATP binding sites, a nucleic acid binding site and a DEAD box helicase motif within the DEXHc_dicer domain; an ATP binding site and a DNA binding site within the Helicase_C domain; an active site, a metal binding site and a dimerization interface of the two ribonuclease III domains; and a putative RNA binding site within the DND1_DSRM domain.

The identified three genes of hops AGO have similar lengths in both transcripts and coding sequences. At the protein level, AGO1 shares 36.65% and 40.29% similarity with AGO2 and AGO7, respectively, while AGO2 shares 38.06% similarity with AGO7. The length of transcripts of hop *AGO1* is 4519 nt, the length of *AGO2* is 4333 nt and the length of *AGO7* is 3485 nt. The length of coding sequences is 1035 a. a., 1038 a. a., and 1029 a. a. for *AGO1*, *AGO2*, and *AGO7*, respectively. However, the *AGO1* gene encompasses 21 introns, while *AGO2* and *AGO7* encompass two introns each. The number of introns of the hop *AGO1* gene is consistent with the number of introns in other closely related species, e.g., *AGO1* of *Cannabis sativa* (XM_030642713.1) and *Ziziphus jujuba* (XM_016028508.2), as well as in more distant species such as *Arabidopsis lyrata* subsp. *lyrata* (XM_021012707.1), with which it shares 98.68%, 95.17%, and 87.33% identity in protein sequence at 100% query coverage, respectively. While the number of introns of hop’s *AGO2* is consistent with the number of introns in the transcripts of species *C. sativa* (XM_030624263.1), *Z. jujuba* (XM_016011349.2) and *A. lyrata subsp. lyrata* (XM_002893803.2), the number of introns of *AGO7* differs between hop (2 introns) and *C. sativa* (3 introns) (XM_030624015.1). However, two introns are annotated in the *AGO7* transcripts of *Z. jujuba* (XM_016039513.2) and in *A. lyrata* subsp. *lyrata* (XM_021035382.1). Although there is a discrepancy in the number of introns of *AGO7* between hop and *C. sativa*, they share 92.37% identity in coding sequences at 100% query coverage. Using the Pfam database, the ArgoN domain (PF16486) at the N-terminal part, followed by Argonaute linker 1 domain (ArgoL1) (PF08699), and the PAZ (PF02170) and PIWI (PF02171) domains at the C-terminal part of the protein sequence were identified in AGO hop proteins. Additionally, a significant match of the glycine-rich region of argonaute (PF12764) was identified upstream of ArgoN domain at the N-terminal part, and the Argonaute linker 2 domain (ArgoL2) (PF16488) and the Mid domain of argonaute (ArgoMid) (PF16487) were identified between the PAZ and PIWI domains in the AGO1 protein sequence of hops. A less significant match with the Pfam database was observed for ArgoL2 domain in AGO2 and AGO7, and ArgoMid domain in AGO7 (Figure 1c–e). Using the conserved domain database, the nucleic acid-binding region was identified within the PAZ domain and the 5’ RNA guide strand anchoring site, and an active site were identified within the PIWI domain of all three hops AGO protein sequences.

*RDR2* gene contains three introns and encodes transcript with 3743 nt in length, which contain coding sequence that is translated into 941 amino acids long polypeptide. Same number of introns can be observed in *RDR2* gene of *C. sativa* (XM_030637773.1) and model plant species *A. thaliana* (NM_117183.3). On the other hand, *RDR6* contains 2 introns with transcript length of 4270 nt and coding sequence translated into 1204 amino acids in length. As for *RDR2*, the number of introns in *RDR6* is same as in *C. sativa* (XM_030645365.1) and *A. thaliana* (NM_001339423.1). Hops RDR2 and RDR6 share 33.40% similarity in protein sequences and no significant similarity at transcript level. According to the Pfam analysis both proteins, RDR2 and RDR6, contain RNA dependent RNA polymerase domain (RdRP; PF05183) at C-terminal part (Figure 1f,g). Additionally, using CDD to predict conserved domains, both proteins contain RNA recognition motif (RRM) also known as RNA binding domain (RBD; cd00590) at N-terminal part.

### 2.2. Phylogenetic Analysis

**Figure 2:**
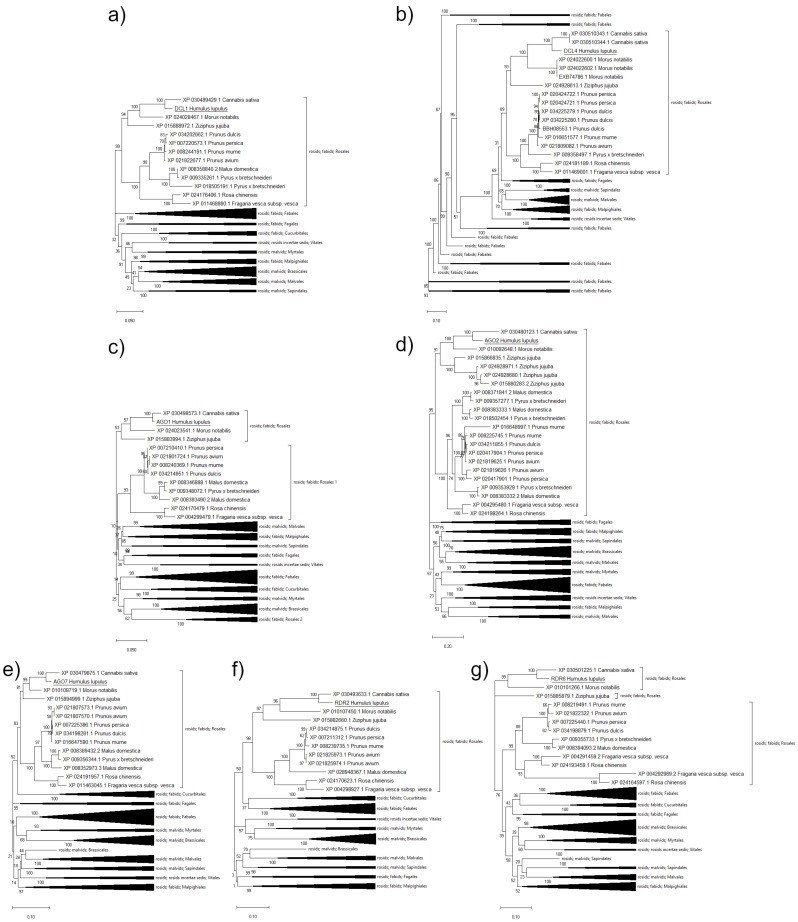
Unrooted phylogenetic trees constructed using the maximum likelihood method (Jones-Taylor-Thornton (JTT) model) based on comparison of amino acid sequences of hops (**a**) DCL1 (MW658774), (**b**) DCL4 (MW658775), (**c**) AGO1 (MW658771), (**d**) AGO2 (MW658772), (**e**) AGO7 (MW658773), (**f**) RDR2 (MW658776) and (**g**) RDR6 (MW658777) and their homologs from species in the clade Rosidae (NCBI: taxid: 71275). Numbers above nodes indicate the reliability of 1000 bootstrap replicates. Scale bar in each panel represents amino acid substitution per site.

A phylogenetic analysis based on evolutionarily conserved proteins involved in the RNAi pathway was performed to determine the phylogenetic relationship between RNAi components of hops and species of the clade Rosids, namely Rosidae (NCBI: taxid: 71275). In the phylogenetic analyses of the identified RNAi proteins, we observed, as expected, that the hop proteins clustered together with proteins of closely related species, i.e., *C. sativa* (Cannabaceae), and form a group with *M. notabilis* (Moraceae) and *Z. jujuba* (Rhamnaceae). The ML trees of all analyzed proteins show that the group comprising hop, *C. sativa*, *M. notabilis* and *Z. jujuba* is closely related to species of the family Rosaceae and we can observe that these two groups form a common cluster of species belonging to the order Rosales (Figure 2). The ML trees show that the species of the same order are clustered together. The DCL1 protein of hop shares 94.71% identity with its homolog from *C. sativa* (XP_030489429.1), 87.92% identity with *M. notabilis* (XP_024028467.1), and 83.99% identity with *Z. jujuba* (XP_015888972.1). With homologs from the family Rosaceae, hops DCL1 shares 80.02% (*Pyrus* x *bretschneideri*; XP_018505191.1) to 83.57% (*Prunus avium*; XP_021822677.1) identity (Figure 2a). Hop DCL4 shares from 64.37% identity (*Rosa chinensis*; XP_024181189.1) to 85.62% identity (*C. sativa*; XP_030510344.1) with species of the order Rosales (Figure 2b). For AGO1, we observed two clades with species from the family Rosaceae, namely Rosales 1 and Rosales 2 (Figure 2c). AGO1 isoforms of *Prunus persica*, *Prunus avium*, *Prunus mume*, *Prunus dulcis*, *Malus domestica*, *Pyrus* x *bretschneideri*, *Malus domestica* and *Fragaria vesca* are present in both clades. However, hop AGO1 shares with isoforms of clade Rosales 1 from 91.62% to 93.81% identity, while with isoforms of clade Rosales 2 from 85.02% to 88.53% identity (Figure 2c). For AGO2, we observed that the clade with species of the order Rosales comprises two subclades. Hop AGO2 is clustered in the subclade with *C. sativa*, *M. notabilis* and *Z. jujuba*, with which it shares from 58.33% to 76.29% identity in protein sequences. The second subclade contains species of the Rosacea family, with which the hop AGO2 protein sequence shares from 56.36% to 63.73% identity (Figure 2d). A similar clustering is also observed in the ML tree of AGO7. Hop AGO7 has the highest protein sequence identity with AGO7 from *C. sativa* (92.98%), *M. notabilis* (87.88%) and *Z. jujuba* (84.39%), while it shares from 80.28% to 86.63% identity with AGO7 from species of the Rosaceae family (Figure 2e). Sequence identity between hop RDR2 and homologs from the order Rosales ranged from 69.01% (*Fragaria vesca* subsp. *vesca*; XP_004298927.1) to 91.38% (*Cannabis sativa*; XP_030493633.1), while it was generally lower for RDR6, ranging from 62.48% (*Fragaria vesca* subsp. *vesca*; XP_004292989.2) to 89.63% (Cannabis sativa; XP 030501225.1) (Figure 2f,g).

### 2.3. Differential Expression of RNAi Genes

**Figure 3:**
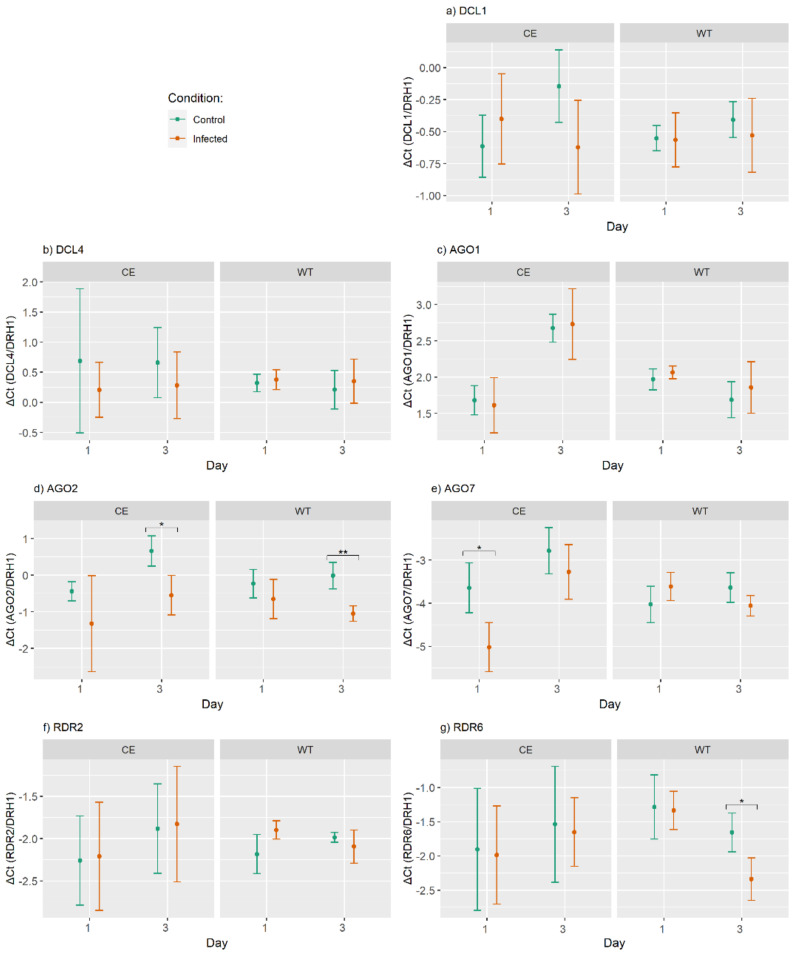
Expression levels of hop RNAi genes (**a**–**g**) in control and *V. nonalfalfae*-infected root tissue of the susceptible cultivar Celeia (CE) and the resistant cultivar Wye Target (WT) on the first and third day post-inoculation. The data are presented as mean ± standard deviation of the ΔC_t_ values. An asterisk “*” indicate significant differences between control and treated samples at a signifi-cance level FDR *p*-value ≤ 0.05, and “**” at FRD *p*-value ≤ 0.01.

Differential expression analysis of selected RNAi core components, *DCL1*, *DCL4*, *AGO1* and *RDR2*, performed upon infection with *V. nonalfalfae* for the susceptible cultivar Celeia and the resistant cultivar Wye Target at 1 dpi and 3 dpi didn’t revealed any differences in the expression between infected and control samples (Figure 3a–c,f).

The expression of *AGO2* was statistically significantly different between *V. nonalfalfae*-infected and control samples at 3 dpi in the susceptible and resistant hop cultivars. In the susceptible cultivar, a 1.2-fold decrease (FDR *p*-value = 0.017) in the expression of *AGO2* was observed in infected samples (−0.54 ± 0.54) compared to the controls (0.66 ± 0.42). Similarly, in the resistant cultivar 1-fold decrease (FDR *p*-value = 0.002) was observed in infected samples (−1.1 ± 0.21) as compared to control samples (−0.01 ± 0.36) (Figure 3d). 

*AGO7* showed significantly altered expression in response to infection with *V. nonalfalfae* in the susceptible cultivar at 1 dpi. Its expression was 1.4-fold lower (FDR *p*-value = 0.043) in the infected samples (−5.02 ± 0.57) compared to the controls (−3.64 ± 0.58) (Figure 3e). In the resistant cultivar, we observed a significant 0.7-fold decrease (FDR *p*-value = 0.021) in the expression of *RDR6* between infected (−2.34 ± 0.31) and control (−1.66 ± 0.28) samples at 3 dpi (Figure 3g).

## 3. Discussion

The RNAi mechanism is referred to as a conserved gene silencing process mediated by small RNAs and RNAi components. It is considered to be one of the most important mechanisms in the regulation of gene expression as well as in the defense response of plants to various phytopathogens, i.e., silencing of exogenous RNAs. In our study, core RNAi components of hops involved in miRNA and ta-siRNA RNAi pathways were identified and characterized at the protein domain level for the first time. Obtaining expression profiles of RNAi core components in susceptible and resistant hop cultivars would enable us to know which RNAi pathways play an important role during Verticillium wilt pathogenesis and which physiological processes are affected by the RNAi mediated response to fungal infection.

In plants, there are several pathways of RNA interference dictated by different sRNAs originating from the activity of specific DCL proteins. DCL1 is involved in the biogenesis pathway of microRNAs (miRNAs) by cleaving primary miRNAs (pri-miRNAs) and precursor miRNAs (pre-miRNAs) to produce miRNAs [5]. DCL1 is also indirectly involved in the biogenesis of ta-siRNAs by participating in the production of miRNAs that initiate ta-siRNA production [6,8]. DCL1-produced mature miRNAs are loaded into the AGO1 protein to form RISC, which binds to complementary sequences in mRNAs and represses their translation into proteins [11]. 

Phylogenetic analysis performed on a group of species in the clade Rosidae (NCBI: taxid: 71275) confirmed the relatedness of hop RNAi proteins to corresponding orthologs [29,30]. The hop proteins clustered together with proteins from closely related species, i.e., *C. sativa*, forming a group with *M. notabilis* and *Z. jujuba*. Comparing hop to the closest related species, *C. sativa,* we observed fewer amino acid substitutions per site in all analyzed hop RNAi core components, except for RDR2 (Figure 2). The cluster comprising hops, *C. sativa*, *M. notabilis,* and *Z. jujuba* is closely related to other species of the family Rosaceae which belong to the order Rosales, and the same pattern of clustering is also observed by Zhang et al. [30]. The RNAi core components analyzed in our study exhibit highly conserved gene structures, resulting in conserved number of introns among hops and closely related species, i.e., *C. sativa*, *M. notabilis,* and *Z. jujuba*, and also *Arabidopsis*. The highest similarity of amino acid sequences between hops, *C. sativa*, *M. notabilis* and *Z. jujuba* was observed for AGO1 (98.7%, 94.7%, 95.2%, respectively), followed by DCL1 (from 83.9% to 94.7%), AGO7 (from 84.4% to 92.9%), RDR6 (from 75.2% to 89.6%), RDR2 (from 73.5% to 91.4%), DCL4 (from 68.2% to 85.6%), AGO2 (65.1% to 76.3%). Based on our results, we can conclude that core components of the miRNA-mediated RNAi pathway (DCL1 and AGO1) might be more conserved among hops and its closely related species than RNAi components of the siRNA-mediated pathway (DCL4, AGO2, AGO7, RDR2, and RDR6). Moreover, the characterization of protein domains and the results of the phylogenetic analysis suggest that protein structures are also highly conserved among hops, *C. sativa*, *M. notabilis* and *Z. jujuba*, and even across species from the order Rosales, but distinct from species of other orders. The high degree of conservation may reflect the selection pressure exerted by the essential functions of RNAi, as this mechanism is one of the most important biological processes in plant species.

In our study, we did not observe altered expression of *DCL1* or *AGO1*, which play a role in miRNA biogenesis and PTGS mediated by miRNAs. However, in two grapevine (*Vitis vinifera*) cultivars, the disease-resistant cultivar ‘Norton’ and the susceptible cultivar *Cabernet sauvignon*, the researchers observed altered expression of *DCL1* after inoculation with fungus *Erysiphe necator*, the causal agent of powdery mildew in grapes. In both cultivars, *DCL1* exhibit a similar expression pattern and the peak of *VvDCL1* expression was observed at 4 h post-inoculation, followed by a gradual decrease from 8 h to 12 h and gradual increase until 48 h post-inoculation. At 4, 8 and 24 h after infection, the researchers observed relatively higher expression levels of *VvDCL1* in the susceptible cultivar *Cabernet sauvignon* compared to the expression levels observed in the resistant cultivar Norton [31]. Given the dynamics of *DCL1* expression in grapevine infected with *E. necator*, where expression differences were observed within 24 h [31], we could assume that in our experiment there was no noticeable difference in *DCL1* expression due to the sampling time points which were in our case 1 and 3 dpi. Furthermore, *DCL1* shows differential expression in roots and shoots of *Arabidopsis* plants exposed to various abiotic stress factors, such as drought, cold and salt [31], while none of *DCL* genes identified in *O. sativa* showed differential expression in response to cold, salt or dehydration [32]. Decreased expression of *DCL1* was observed in rice infected with the rice blast fungus (*Magnaporthe oryzae*) from 0 to 72 h after inoculation, and silencing of *OsDCL1* enhances basal resistance to the fungus probably due to increased expression of defense related genes that are regulated by DCL1-produced miRNAs [33]. DCL1 also mediates processing of DNA virus-derived small interfering RNAs and DNA virus-induced silencing [34]. Altered expression of *DCL1* was observed in Fibermax (virus-susceptible) and Delta Opal (virus-resistant) cotton plants after infection with *Cotton leafroll dwarf virus* (CLRDV). At 1 dpi, the susceptible cultivar Fibermax showed significantly lower expression of *DCL1* compared to the virus-resistant cultivar Delta Opal, however, significantly higher expression of *DCL1* was observed in the susceptible cultivar at 5 and 15 dpi compared to the resistant cultivar [35]. On the other hand, *DCL1* showed no differential expression in leaves, flowers or cones of hop cultivar Celeia infected with hop latent viroid or citrus bark cracking viroid [28]. 

AGO1 is major AGO protein, which associates with DCL1-produced miRNAs (21-nucleotide in length and 5′ terminal uridine) and dictates miRNA-directed PTGS. AGO1 also associates with 22-nucleotide miRNAs to trigger biogenesis of secondary siRNAs (ta-siRNAs) [11,12]. Knockout of *ago1* increases resistance to *V. dahliae* [27] and *B. cinerea* [25] in *Arabidopsis* and *V. longisporum* in *B. napus* [36]. In contrast, *Arabidopsis ago1* mutants were more susceptible to infection with *S. sclerotiorum*, while *ago1* overexpression led to increased resistance [26]. Based on these studies, we can observe that the role of AGO1 may be specific to a particular plant species and its interaction with the phytopathogen. Moreover, the RNAi defense response mediated by the DCL1-AGO1-miRNA module may be specific to different pathosystems. The extent to which the pathogen induces perturbations in the expression of *DCL1*, *AGO1*, and miRNAs may affect host growth and development, as the module plays a critical role in those processes [32,37,38]. The homeostasis of plant DCL1 and AGO1 is regulated by miR162 and miR168, respectively [39,40]. In our previous study we identified hop miRNAs that respond to infection with *V. nonalfalfae* at 1 dpi (under review; https://doi.org/10.21203/rs.3.rs-114355/v1; accessed on 16 April 2021) and we did not detect changes in the expression of hlu-miR162 or hlu-miR168, which may explain why we did not observe changes in the expression of *DCL1* or *AGO1*. The results of our study suggest that in hops the initial stages of the Verticillium wilt pathogenesis do not induce changes in the regulation of gene expression that would stop the processes of growth and development. 

Like AGO1, AGO2 is also involved in miRNA- or siRNA-directed PTGS. AGO2 associates mainly with 21-nucleotide long sRNAs with adenosine at the 5’ end [13]. Both *AGO1* and *AGO2* showed increased expression in response to *S. sclerotiorum* infection in leaves of *B. napus* [26]. AGO2 is involved in the innate immunity of *Arabidopsis* plants challenged with the bacterial pathogen *Pseudomonas syringae* pv. *tomato* (*Pst*), and the study showed that AGO2 protein levels in leaves were strongly induced by the pathogen [41]. Moreover, plant AGO2 also plays a role in antiviral RNA silencing due to its ability to load Cucumber mosaic virus (CMV)-derived siRNAs [42]. In contrast to the results of the aforementioned studies, where plants are challenged by bacterial or viral phytopathogens, the results of our study show downregulation of *AGO2* in the roots of susceptible and resistant hop cultivars on the third day after inoculation with the fungal pathogen *V. nonalfalfae*. The contrary results suggest that AGO2 has distinct roles in host defense against bacteria, viruses, and fungi [31,41,42], although its role in response to fungi remains to be elucidated. It has been suggested that AGO2 also binds ta-siRNAs [13], therefore downregulation of *AGO2* in hop plants infected with *V. nonalfalfae* may contribute to reduced activity of ta-siRNAs involved in the defense response against the fungus. 

We also examined the expression of the major components involved in ta-siRNA biogenesis, i.e., *DCL4*, *RDR6* and *AGO7*. We did not detect altered expression of *DCL4* in either hop cultivar at any sampling point. However, we detected significant downregulation of *AGO7* in the susceptible cultivar Celeia at 1 dpi and significant downregulation of *RDR6* in the resistant cultivar Wye Target at 3 dpi. In our study of hop *V. nonalfalfae*-responsive miRNAs at 1 dpi (under review), we detected upregulation of hlu-miR390a in the susceptible cultivar Celeia (log_2_FC = 1.87, *p*-value = 0.02). In *Arabidopsis*, miR390 and AGO7 form a miRNA-guide/effector protein pair which guides the cleavage of *TAS* transcripts, which are further reverse transcribed by RDR6 to longer dsRNAs. The latter are processed by DCL4 into 21-nucleotide duplexes and one of the two strands of each duplex (ta-siRNA) is loaded into the AGO to drive PTGS of the target transcripts [7,12]. Thus, the accumulation of hlu-miR390a in the roots of the susceptible cultivar Celeia after infection with *V. nonalfalfae* could be due to downregulated AGO7, which in turn suppresses the ta-siRNA biogenesis pathway. Ellendorff et al. [27] found that *Arabidopsis ago7* mutants were more susceptible to infection by *V. dahliae* and showed severe disease symptoms compared to inoculated Col-0 plants. This suggests that AGO7 may play an important role in the pathogenesis of Verticillium wilt, probably through the ta-siRNA production or some other defense mechanisms mediated by siRNAs, which remains to be elucidated. However, in the resistant cultivar Wye Target, it can be assumed that the biogenesis of ta-siRNAs was suppressed at 3 dpi by downregulation of *RDR6*, which is involved in the process of converting transcripts to dsRNAs, which are subsequently processed by DCL4. Since ta-siRNA biogenesis of infected plants of both cultivars was suppressed in either way (silencing of AGO7 or RDR6), *DCL4* did not show altered expression between infected and control samples. In *Arabidopsis*, RDR6 together with AS1 (ASYMMETRIC LEAVES1) and AS2, regulates plant development by repressing miR166 [43]. Overexpression of miR166 in the root apical meristem (RAM) negatively regulates transcripts of the class III HD-ZIP family and thus promotes the activity of RAM [44,45]. In our case, the downregulation of *RDR6* in the resistant cultivar Wye Target at 3 dpi could affect the production of ta-siRNA and, on the other hand, allow the expression of miR166 (no data available for miR166 expression at 3 dpi), which consequently downregulates the transcripts of the class II HD- ZIP (homeobox-leucine zipper protein hdg11) gene, thus promoting root development during *V. nonalfalfae* infection. In the transcriptomic study, Progar et al. [46] observed a −2.3-fold decrease in transcripts of the HD-ZIP family in the resistant cultivar Wye Target at 6 dpi after infection with *V. nonalfalfae*, which was not observed in the susceptible cultivar Celeia. Moreover, most of the genes that showed altered expression in the resistant cultivar were related to secondary metabolism and lateral root development [46].

It can be observed that both cultivars silence ta-siRNA biogenesis to some extent, but in different ways and at different time points after infection. Since both *Arabidopsis ago7* and *rdr6* mutants showed enhanced symptoms upon *Verticillium* infection [27], it can be hypothesized that downregulation of *AGO7* in the susceptible cultivar Celeia at 1 dpi may result in reduced PTGS with other AGO7-associated sRNAs, which in turn may be reflected in increased susceptibility and symptoms severity of the cultivar Celeia. In contrast, in the resistant cultivar Wye Target, reduced ta-siRNA-mediated regulation due to downregulated *RDR6* may lead to constitutive defense against the fungus via the formation of lateral roots and activation of secondary metabolite biosynthesis, as observed in previous studies [46,47]. The results of our study suggest that *AGO7* in the susceptible hop cultivar and *RDR6* in the resistant hop cultivar may represent novel targets for genetic improvement. Overexpression of *AGO7* in the susceptible hop cultivar may result in increased tolerance of the plant to Verticillium infection. Similarly, downregulation of *RDR6* and, in turn, increased miR166 activity may induce increased root apical meristem activity, thereby increasing the plant’s tolerance to fungal infection. 

In the future, miRNA sequencing of samples from other sampling points should be performed to determine the temporal expression of miRNAs and integrate the expression profiles of miRNAs, their corresponding targets, and RNAi component to elucidate the role of RNAi in hop defense mechanisms against *V. nonalfalfe*.

## 4. Materials and Methods

### 4.1. Identification of RNAi Genes

Computationally assembled transcriptome data of hop is available from our laboratory [28]. The raw NGS sequences are publicly available under BioProject number PRJNA342762, BioSample SAMN05767836, SRA run SRR4242068. To build and manually curate gene models of hop RNAi core components, we downloaded the amino acid sequences of AGO, DCL, and RDR, which belong to the species of the clade Rosidae (NCBI: taxid: 71275), from the UniProtKB database (https://www.uniprot.org/; accessed on 16 April 2021). Hop transcriptome data were aligned against obtained amino acid sequences using the BLASTX algorithm implemented in the CLC Genomic Workbench (ver. 11.0.1) (QIAGEN Digital Insights, Aarhus, Denmark), and the hits with highest *e*-values and >95% coverage were selected for further analysis. To identify *AGO*, *DCL* and *RDR* genes in hop draft genome, identified homologs in transcriptome and obtained homologs from UniProtKB database were aligned against hop draft genome sequences using BLAST and tBLASTn algorithms implemented in the CLC Genomic Workbench (ver. 11.0.1) (QIAGEN Digital Insights, Aarhus, Denmark), respectively. Genome regions to which the transcript and homolog of corresponding RNAi protein from the UniProtKB database aligned were used to construct gene models. Furthermore, hops RNA-Seq data were mapped against hops genome with the Large Gap Read Mapping tool implemented in the CLC Genomic Workbench (ver. 11.0.1) (QIAGEN Digital Insights, Aarhus, Denmark) to support manual curation of gene models. The protein coding sequence within transcripts was predicted using NCBI ORFfinder (https://www.ncbi.nlm.nih.gov/orffinder/; accessed on 16 April 2021). Thus obtained protein sequences were further searched for the protein functional domains using Pfam 33.1 (https://pfam.xfam.org/; accessed on 16 April 2021) and CDD v3.18 (https://www.ncbi.nlm.nih.gov/Structure/cdd/wrpsb.cgi; accessed on 16 April 2021) web-based tools. Characteristic domains were searched for all three groups of proteins. AGO proteins had to contain AgoN (PF16486), PAZ (PF02170), and PIWI (PF02171) domains. DCL proteins were searched for DExD-box (PF00270), Helicase-C (PF00271), PAZ (PF02170), ResIII (PF00636), and DND1_DSRM (PF14709) domains. RdRP domain (PF05183) was searched within RDR protein sequences. The theoretical pI (isoelectric point) and Mw (molecular weight) were calculated using ExPASy Compute pI/MW online tool (https://www.expasy.org/resources/compute-pI-mw; accessed on 16 April 2021). 

### 4.2. Phylogenetic Analysis

Identified proteins of AGO, DCL, and RDR in hops and retrieved amino acid sequences of species of the clade Rosidae (NCBI: taxid: 71275) from the NCBI reference proteins database were used in phylogenetic analysis performed in MEGA 10.0.5 [48]. In a first step the multiple sequence alignment of the amino acid sequences of each identified protein in hops and its homologs was performed with the MUSCLE algorithm implemented in MEGA 10.0.5. The phylogenetic analysis was performed with maximum likelihood method and Jones–Taylor–Thornton (JTT) model. In the analysis, a partial deletion of sites with a site coverage cutoff at 95% was set. The reliability of the tree nodes was tested with 1000 bootstrap replicates. 

### 4.3. Hop Inoculation Experiment

The artificial inoculation was performed on plants of two hop cultivars; Celeia which is susceptible to Verticillium wilt and Wye Target that has introgression of Verticillium wilt resistance from US germplasm (detailed description in Jakse et al. [49]). Hop plants were provided by the Slovenian Institute of Hop Research and Brewing where they propagate plants as softwood cuttings in a greenhouse or as dormant cuttings from the rootstock. One-year old rooted cutting were used in the experiment. For each sampling point (1 dpi and 3 dpi), the roots of three biological replicates of the susceptible or resistant hop plants were immersed for 10 min in a suspension containing conidia of the highly virulent strain of *V. nonalfalfae* (PV1, isolate T2) (5 × 10^6^ conidia/mL), as proposed by Flajsman et al. [50], and three biological replicates of each cultivar were mock-inoculated using sterile water (controls). The roots of *V. nonalfalfae*-inoculated and control plants were sampled at one day and three days post-inoculation. The roots were cut off the bines, washed, freeze-dried with liquid nitrogen in a pre-cooled mortar and ground to a fine powder. Samples were stored at −80 °C until isolation of total RNA. The presence of fungal DNA in infected plants and absence of fungal DNA in control plants was confirmed as described in Kunej et al. [51].

### 4.4. Total RNA Isolation and qPCR Analysis

Total RNA was isolated from 100 mg root tissue using Spectrum™ Plant Total RNA Kit (Sigma-Aldrich, St. Louis, MI, USA) according to manufacturer’s instructions. The concentration and quality of the extracted RNA were assessed with Bioanalyzer Agilent^®^ RNA6000 Kit and Agilent^®^ 2100 Bioanalyzer^®^ instrument (Agilent Technologies, Inc., Santa Clara, CA, USA) following the manufacturers’ instructions. First strand cDNA synthesis was performed using High-Capacity cDNA Reverse Transcription Kit (Applied Biosystems, Foster City, CA, USA) according to manufacturers’ instructions. Specific primers for each gene of interest were designed using Primer3 online tool (https://bioinfo.ut.ee/primer3/; accessed on 16 April 2021) (Supplementary Table S1), checked for specificity in binding using Primer-BLAST (https://www.ncbi.nlm.nih.gov/tools/primer-blast/; accessed on 16 April 2021) and analyzed using OligoAnalyzer (https://www.idtdna.com/calc/analyzer; accessed on 16 April 2021). Quantitative RT-PCR was performed with 7500 Real-Time PCR System (Applied Biosystems, Foster City, CA, USA) and Fast SYBR^®^ Green technology (Thermo Fisher Scientific, Waltham, MA, USA). The reaction was performed in a volume of 10 µL containing 5 µL Fast SYBR Green Master Mix, 10 ng of cDNA and 300 nM of each forward and reverse primer. We used following amplification program: 95 °C for 20 s, 40 cycles at 95 °C for 3 s, and 60 °C for 30 s, followed by melt curve stage starting at 95 °C for 15 s, 60 °C for 60 s, 95 °C for 15 s, and 60 °C for 15 s to monitor primer specificity. Previously developed reference gene sequence for DEAD-box ATPase-RNA-helicase (*DRH1*) was used as internal control for data normalization [52]. The amplification efficiency of primers was calculated from 5-fold serial dilution in range from 50 to 0.08 ng/µL with 7500 Software v2.3 (Applied Biosystems, Foster City, CA, USA). The relative expression levels of the genes of interest (GOI) were determined based on the mathematical model proposed by Livak and Schmittgen [53]. For each sample, the cycle threshold value (Ct) of GOI was subtracted from the Ct value of the reference gene to obtain delta cycle threshold value (ΔCt). Prior to the ANOVA analysis, normality was assessed using Shapiro–Wilk’s normality test and Levene’s test was used to estimate the homogeneity of variances. The residuals were normally distributed (*p* > 0.05) and there was homogeneity of variances (*p* > 0.05). One-way ANOVA and Tukey’s post-hoc test were used to analyze differences in the relative amounts of GOIs (ΔCt) between the *V. nonalfalfae*-inoculated and control samples. Statistically significant differences between the samples were tested at a significance level FDR *p*-value ≤ 0.05. Data were analyzed using an R software environment for statistical computing [54] and R package rstatix [55].

## Figures and Tables

**Table 1:** Characteristics of core RNAi genes identified in hops.

Protein	Number of Introns	Transcript Length (nt)	CDS Length (aa)	pI	Mw (Da)
DCL1	19	6497	1984	6.03	222,623.18
DCL4	24	5281	1645	6.14	184,947.75
AGO1	21	4519	1035	9.22	114,542.48
AGO2	2	4333	1038	9.41	115,025.66
AGO7	2	3485	1029	9.33	117,545.61
RDR2	3	3743	941	7.03	106,793.04
RDR6	2	4270	1204	6.66	137,281.43

pI denote isoelectric point of a protein; Mw denote molecular weight of a protein in Daltons (Da).

## Data Availability

Data supporting results are within the paper and its Supplementary Materials. The computationally assembled hops transcriptome is available from our research group and the raw NGS sequences of hops transcriptome are deposited in NCBI’s SRA archive under BioProject number PRJNA342762, BioSample SAMN05767836, SRA run SRR4242068: https://www.ncbi.nlm.nih.gov/sra/?term=SRR4242068 (accessed on 16 April 2021). The hop draft genome was obtained from the HopBase genomic resource which is available at http://hopbase.org (accessed on 16 April 2021) and http://hopbase.cgrb.oregonstate.edu (accessed on 16 April 2021). Transcript and protein sequences (mRNA) were deposited in NCBI’s database with following accession numbers; Argonaute protein 1 (MW658771), Argonaute protein 2 (MW658772), Argonaute protein 7 (MW658773), Dicer-like protein 1 (MW658774), Dicer-like protein 4 (MW658775), RNA-dependent RNA polymerase 2 (MW658776) and RNA-dependent RNA polymerase 6 (MW658777).

## Supplementary Materials

The following are available online at https://www.mdpi.com/article/10.3390/ijms22084224/s1, Table S1: Primer sequences used for RT-qPCR on RNA interference genes.
